# The Polymorphism of Orlyum White 520T, an Ultraviolet Luminescent Security Ink

**DOI:** 10.3390/molecules30081671

**Published:** 2025-04-08

**Authors:** János Madarász, Nóra V. May, Petra Bombicz, György Pokol, Richárd Kocsis, Bálint Hegymegi-Barakonyi, Tibor Gizur

**Affiliations:** 1 Department of Inorganic and Analytical Chemistry, Budapest University of Technology and Economics, Műegyetem rkp. 3, HU-1111 Budapest, Hungary; pokol.gyorgy@vbk.bme.hu; 2 Centre for Structural Science, HUN-REN Research Centre for Natural Sciences, Magyar Tudósok Körútja 2, HU-1117 Budapest, Hungary; bombicz.petra@ttk.hu; 3 LuminoChem R&D Ltd., Háros utca 7/A, HU-1222 Budapest, Hungary; kocsis@luminochem.com (R.K.); barakonyi@luminochem.com (B.H.-B.); 4 Department of Organic Chemistry and Technology, Budapest University of Technology and Economics, Műegyetem rkp. 3, HU-1111 Budapest, Hungary; gizurtibor@gmail.com

**Keywords:** structure–property relationship, ultraviolet (UV) fluorescence, single-crystal and powder X-ray diffraction (XRD), FT-IR spectroscopy, differential scanning calorimetry (DSC), TG/DTA-MS

## Abstract

The polymorphism of the ultraviolet luminescent security ink Orlyum White 520T (*N*-(2-(4-oxo-4*H*-benzo[d][3,1]-oxazin-2-yl)phenyl)naphthalene-2-sulfonamide) is revealed, obtaining two new polymorphic forms with enhanced stability. Beyond the known form (lit. mp. 184.8–185.2 °C, Form III, YOCTAO), we succeeded in gaining two new polymorphic forms, Form II and Form I, with higher melting points of 195–196 and 197–198 °C, respectively. Their elemental composition, ^1^H and ^13^C NMR spectra have been found to be identical, while their powder XRD patterns and FT-IR spectra are significantly different. Based on the single-crystal structure determination of Form II and redetermination of Form III, we uncover the similarities and differences in their packing arrangement and in their secondary interaction systems, all of which affect the molecular conformations in their crystals. In order to explain their significantly distinguishable melting points, Hirshfeld surface analysis and lattice energy calculations have also been carried out. We have made efforts toward revealing the reproducible conditions under which certain polymorphs are formed. It seems that the solvents or other probable organic contaminations are more likely responsible for the formation, nucleation and growth of crystals of various polymorphic forms, traced by thermogravimetric evolved gas analysis (TG/DTA-EGA-MS).

## 1. Introduction

Various derivatives of 2-(2′-arylsulfonylaminophenyl)-4*H*-3,1-benzoxazin-4-ones (Table 1) usually have fluorescent properties, which may be useful, for example in the preparation of security inks or in various applications of fluorescence. Up to August 2024, a total of 122 substituted derivatives of the compound 2-(2′-arylsulfonylaminophenyl)-4*H*-3,1-benzoxazin-4-one were found to be mentioned in the chemical literature [1]; among them, the preparations of 95 substances are described (57 in detail) and the use of 51 (technical or engineering use of 45) are mentioned. A considerable number of these preparations, as well as the physical characterizations of the title compound, were performed first by a Russian scientist, B. M. Bolotin (All-Union Scientific Research Institute of Chemical Reagents and Ultrahigh Purity Chemical Substances, Moscow, USSR) [2]. In Table 1, we summarized, that there are nine crystalline derivatives of the title compound, those were mentioned along with their experimental melting points [1], and there are seven substances whose atomic coordinates are available in the Cambridge Structural Database [3,4]. Two of them have both the structure and the melting point determined, namely 2-(2′-tosyl-aminophenyl)-4*H*-3,1-benzoxazin-4-one, (**1b** or **2a**, its CSD refcode is TAPBZO) [5]) and 2-(2′-β-naphthylsulfonylaminophenyl)-4*H*-3,1-benzoxazin-4-one (**1c** or **2f**, YOCTAO) [6]).

The first description of synthetic preparation of our chosen luminophore title compound 2-(2′-β-naphthylsulfonylaminophenyl)-4*H*-3,1-benzoxazin-4-one (**1c** or **2f**), a commercially available security ink member of this family, was published by Bolotin et al. [2] as a one-pot method in pyridine and alcohol added, what was also repeated in related patent applications [17,18]. The reported melting points were 184.8–185.5 °C (recrystallized from acetone) in each publication. Cevasco [19,20] also prepared a sample in basically the same way, but with an increased melting point of 194.5–195.5 °C (from chloroform-hexane). Than Bratscley et al. [21] reported the same substance prepared using a two-step procedure, with a melting point of 195–196 °C (from dichloromethane-ethylacetate) and IR (KBr disc) absorption bands of 1755, 1595, 1335, 1240, 1150 and 650 cm^−1^. Later on Gizur et al. [22] announced a novel three-step synthetic procedure, which results in products with melting points of 192–195 °C; nevertheless, depending on the final reagent and crystallization solvent type, it seems to have two slightly different fluorescent emission spectra and fluorescent colors. Applying acetic acid anhydride resulted in a slightly yellowish UV-emission spectral color (Form I), while from any of the other aprotic solvents (e.g., CH_2_Cl_2_, toluene, acetone, etc.), a slightly greenish (Form II) occurred as a result of 366 nm UV excitation.

The single-crystal structure of this compound recrystallized from acetic acid had already been published in 2012 (CSD code YOCTAO [6]) for space group No. 4 (*P*2_1_) at T = 173 K. In fact, this structure was not publicly available in the Cambridge Structural Database (CSD) until 2019; our first structure determination preceded it in 2017 at 103 K. The corresponding melting point of the compound had previously been reported as 184.5–185.5 °C [2]. The green fluorescence emission spectrum of the compound crystallized from acetic acid with λ_max_ = 520 nm was also observed [6]. (Note. A solvatomorphic form of the title compound, a hemi-*p*-xylene solvated form (YOCSUH [6], Table 1, **2g**), was also found to crystallize, of space group No. 2 (*P*-1) and with yellowish fluorescent spectral maxima at λ_max_ = 540 nm.)

Taking into consideration the existence of our title compound in new solid forms of yellowish fluorescence color, our research aim is to fully characterize and compare the various occurring polymorphic forms of this β-naphthyl sulfonamide derivative, “Orlyum White 520T”, through structural and analytical methods, such as single-crystal and powder XRD, differential scanning calorimetry (DSC), FT-IR and UV-fluorescence spectroscopy.

## 2. Results and Discussion

### 2.1. Relation Among Unreported New Forms I and II and the Old Form III

**Scheme 1:**
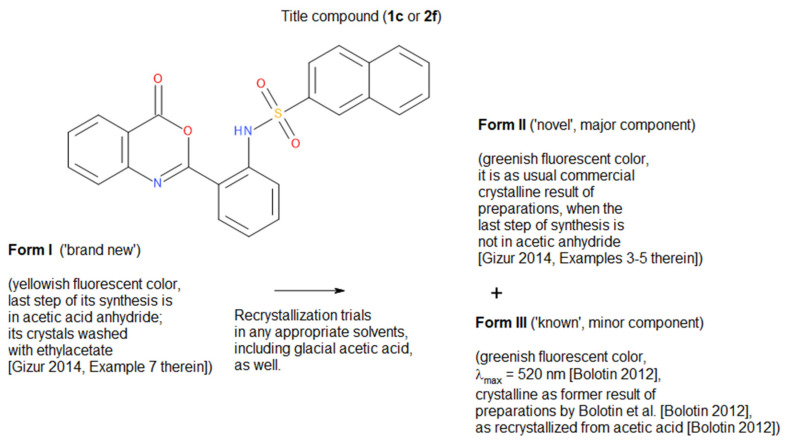
Relation of novel (Form I and Form II) and already-known (Form III) crystalline polymorphic forms of *N*-(2-(4-oxo-4*H*-benzo[d]1,3oxazin-2-yl)phenyl)naphthalene-2-sulfonamide. A crystalline mixture of Form II and III concomitantly forms from Form I during recrystallization trials, independently on the solvent(s) applied. ([Gizur 2014] = [22], [Bolotin 2012] = [6]).

Powder samples of unknown new forms, ‘Sample 1 (Form I)’ and ‘Sample 2 (Form II)’ of *N*-(2-(4-oxo-4*H*-benzo[d]1,3oxazin-2-yl)phenyl)naphthalene-2-sulfonamide (**1c** or **2f**, Scheme 1), were received from Első Vegyi Industria ZRt (Hungary, Budapest). The powder ‘Sample 1 (Form I)’ is a crude product of the final synthesis step (a condensation reaction step) carried out in acetic acid anhydride [22] (Example 7 therein). ‘Sample 2 (Form II)’ is a crude product of the final synthesis step (a condensation reaction step with acetyl chloride) carried out in dichloromethane [22] (Example 3 therein). 

These powder samples, ‘Sample 1 (Form I)’ and ‘Sample 2 (Form II)’, were dissolved in boiling glacial acetic acid or toluene solvents, respectively, to obtain single crystals after cooling. From Form I, we unexpectedly obtained a crystalline mixture of other novel form, Form II and the old one, Form III (Scheme 1). The structure of the harvested single crystal of the already-known Form III was determined at a low temperature of −103 K. Unfortunately, as shown by several additional trials, the recrystallization of ‘Sample 1 (Form I)’ from appropriate solvents, including glacial acetic acid, generally results in a concomitant mixture of the two other polymorphs (Form II and Form III). 

In the case of ‘Sample 2 (Form II)’, single-phase crystals of Form II were grown as expected, i.e., the recrystallization of ‘Sample 2 (Form II)’ showed no change in its polymorphic form; it resulted in a crystalline phase corresponding to the novel Form II, from which single crystals of Form II could be harvested for structure determination.

Unfortunately, homogeneous pure powder samples of form III could not be obtained according to either the synthesis routes of ref. [22] or in other ways. Even commercially available samples purchased from Chemenu Inc. (Shanghai, China, Cat. No. CM140650) have also been proven to correspond to the Form II phase by powder XRD. 

Unfortunately, no single crystal of Form I could be obtained. The recrystallization of ‘Sample 1 (Form I)’ resulted in a mixture of the other two polymorphic forms (as a major phase of Form II and a minor phase of Form III, by powder XRD, Scheme 1).

### 2.2. Identical Composition of Form I and II Proven by CHN(S) Elemental Analysis and NMR Spectroscopy

Elemental analysis showed the following wt% of carbon, hydrogen, nitrogen and sulfur content, as values of two parallel measurements: C% 65.02, 64.81, H% 3.49, 3.59, N% 6.18, 6.30, and S% 7.37, 7.00 for Sample 1 (Form I); C% 65.25, 65.15, H% 3.42, 3.47, N% 6.26, 6.17, and S% 7.30, 7.30 for Sample 2 (Form II). The expected theoretical values are C% 67.28, H% 3.76, N% 6.54, and S% 7.48 for pure title compound C_24_H_16_N_2_O_4_S (CAS Reg. No. 10128-55-9). Comparison indicates the two powder samples have almost the same elemental compositions, slightly deviating from the theoretical one, due to some measurement bias.

The expected 15 aromatic ^1^H NMR signals of Sample 1 (Form I) and Sample **2** (Form II) show the same spectral patterns, considering the identical chemical shift values and couplings and overlaps in the δ = 7–8.5 ppm spectral region, while the aliphatic regions indicate some contaminations, but only to an extent of less than 3–5%. The signal of the fully D-exchangeable NH proton is observed only at around δ = 1.7 ppm (as HOD). The observed 23 out of 24 expected ^13^C NMR signals of Form I and II are practically identical and are summarized in Table 2. (Peak Nos. 12 and 13 are not resolved in either of cases). Due to a lack of pure bulk samples, no data are available on the spectrum of Form III.

### 2.3. Similarities and Differences in Single-Crystal Structures of Form II and III

**Figure 1:**
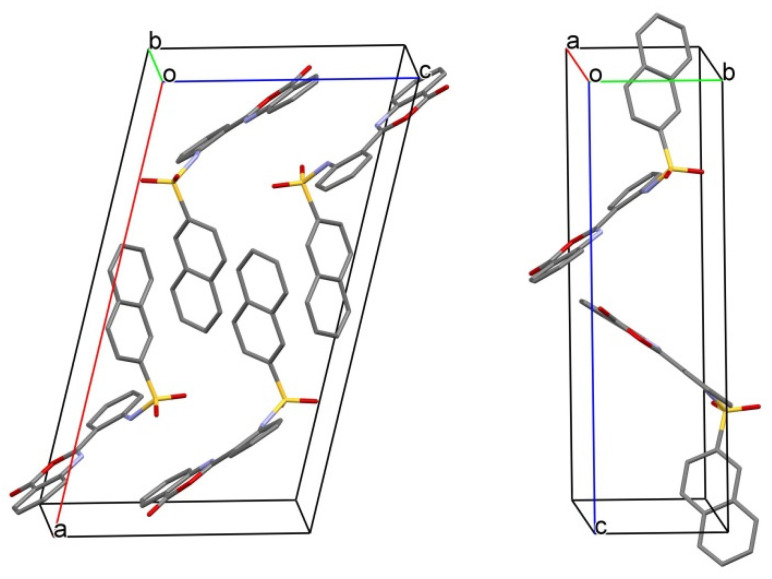
Unit cell of Form II (**left**) and Form III (**right**) of *N*-(2-(4-oxo-4*H*-benzo[d]1,3oxazin-2-yl)phenyl)naphthalene-2-sulfonamide.

We have luckily obtained single crystals for two of the three polymorphic forms of the investigated compound. Unfortunately, single crystals of Form I could not be obtained, and recrystallization turned instead into a mixture of the two other forms (Scheme 1). Form II is found to be a new polymorphic form crystallizing in the centrosymmetric *P*2_1_/c space group. Form III, identical to YOCTAO, crystallizes in the space group *P*2_1_. There is one molecule in the asymmetric unit in both crystal structures. The unit cells of Form II and III are shown in Figure 1, and the packing arrangements from the a, b and c crystallographic directions are shown in Figure S1.

The lengths of the two crystal axes are close to each other (axes b = 8.8822(4) Å and a = 20.215(1) Å of Form II are similar to axes a = 8.7804(7) Å and c = 19.348(2) Å of Form III), while the third one is halved in Form III (b = 5.461(6) Å) in comparison with c = 11.0016(4) Å of Form II; see Table 3. The cell volume of Form III is almost perfectly half that of Form II (960.06(16) Å^3^ and 1919.91(15) Å^3^, respectively). Thus, the crystal density of the two crystals is equal within the calculation error (1.482 mg/cm^3^), and the packing coefficients are equally high (71.3%). There is no residual solvent accessible void in the crystal lattices.

**Figure 2:**
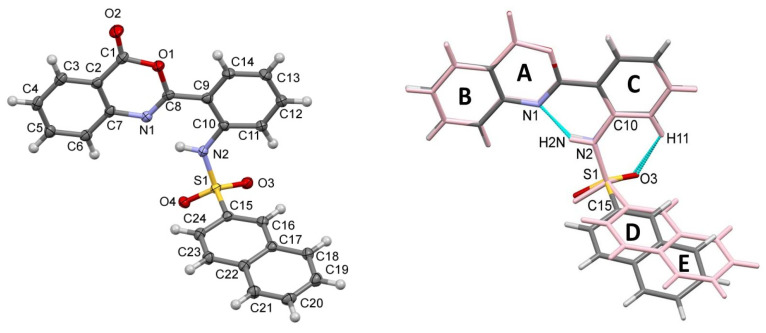
ORTEP diagram of Form II at 50% probability level with atom labels (**left**) and conformational comparison of Form II (colored by element) and Form III (pink) overlaid by their ABC rings (**right**) of *N*-(2-(4-oxo-4*H*-benzo[d]1,3oxazin-2-yl)phenyl)naphthalene-2-sulfonamide.

**Figure 3:**
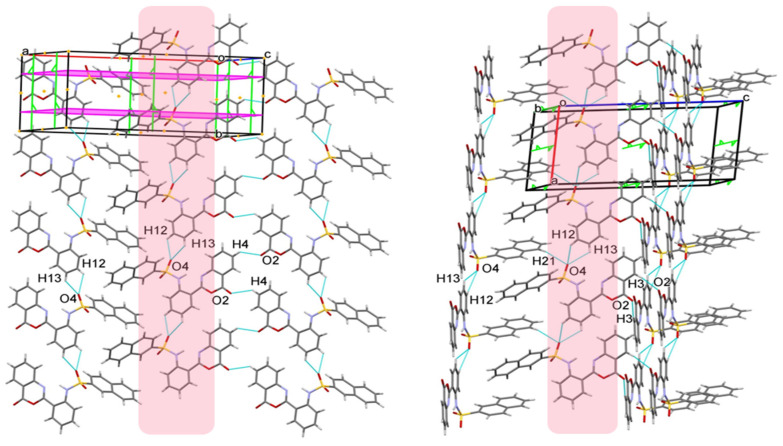
The arrangement of the molecules in the crystal of Form II (*P*2_1_/*c*) along the b = 8.8824(4) Å axis showing the symmetry elements (glide planes are purple, twofold screw axes are green) (**left**), and the arrangement of the molecules in the crystal of Form III (*P*2_1_) along the a = 8.7804(7) Å axis with indication of the symmetry elements, the twofold screw axes (green) (**right**). The highly similar molecular chains formed by the intermolecular interactions in both crystals are highlighted by pink.

The ORTEP representation with atom numbering together with the conformational comparison of the molecules in the two crystal forms are shown in Figure 2. The angle between the benzoxazin (AB) and the phenyl (C) rings deviates only slightly, 2.32(7)° in Form II and 3.6(2) in Form III; however, the C10-N2-S1-C15 torsion angles (which are 72.10(16)° and 62.4(5)°, respectively) differ considerably in the two conformations. Furthermore, the naphthalene ring (DE) is rotated around the S1-C15 single bond in a way that the angles between the planes of ring C and DE are 79.47(7)° and 87.0(2)° in Form II and III, respectively. In both crystals, four intramolecular hydrogen bonds stabilize the conformation, the angle of the AB and the phenyl C rings, e.g., N2-H2N…N1 and C11-H11…O3 (see Figure 3 and Table 4). The N2-H…N1 intramolecular H-bond is a common feature in other related structures as well, which results in the almost planar arrangement of the benzoxazin and the phenyl rings. The average N1…N2 distance calculated for structures TAPBZO, BEHHOL10, TSBZON01, TSBZOX and YOCTAO (see Table 1) is 2.66 ± 0.02 Å; our measured values of 2.654(2) and 2.665(7) Å for Form II and Form III fall into this region. An intramolecular interaction between O1…H14-C14 and O3...H16-C16 further helps to stabilize the conformation.

**Figure 4:**
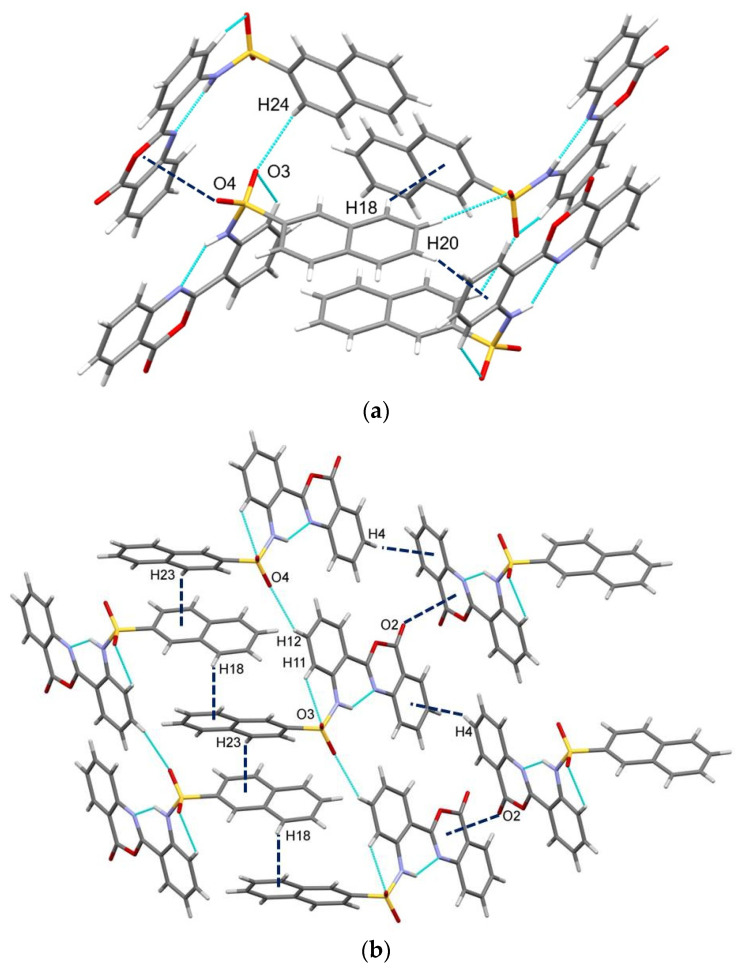
Molecular sheet-type arrangement in the crystal of (**a**) Form II and (**b**) Form III viewed from the ac crystallographic direction. The blue lines show the more significant hydrogen bonds.

The arrangement of the molecules is preserved in both lattices along the 8Å long axes (Figure 3) irrespective of the space groups, owing to the same hydrogen bond systems formed by C12 H12…O4 and C13 H13…O4 interactions (Table 4). Regardless, in the other two dimensions, the placements of the molecular columns are different according to the different crystal symmetries (Figure 3). The determining secondary interactions are hydrogen bonds and X-Y…π interactions in both crystals, but their arrangements are different. Some selected bond distances and angles are collected in Table 4. The significant X-Y…π interactions are shown in Figure 4.

**Figure 5:**
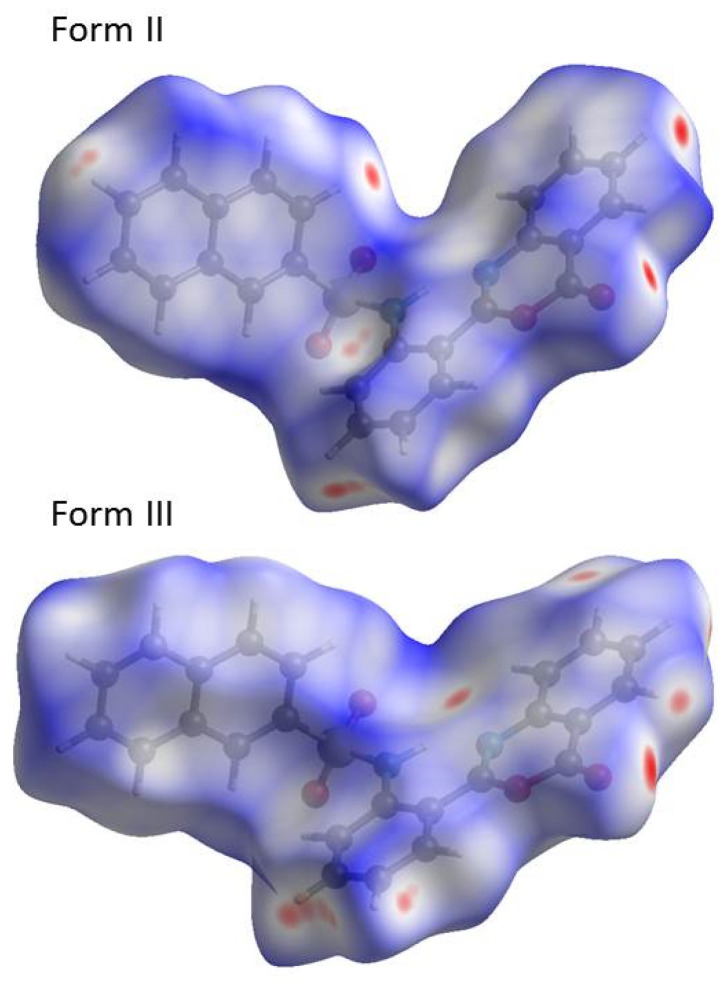
Comparison of the Hirshfeld surfaces of Form II and III (mapped with d_norm_) of *N*-(2-(4-oxo-4*H*-benzo[d]1,3oxazin-2-yl)phenyl)naphthalene-2-sulfonamide. The red spots are showing close contacts with neighboring molecules.

The Hirshfeld surfaces of the molecules in the polymorphic crystals of Forms II and III are illustrated in Figure 5. The transparent presentation allows us to visualize these very similar molecular conformations yet different surfaces of electron density contributions, owing to the different intermolecular interactions in the two crystals. The red spots visible on the surfaces are indicative of close contacts with neighboring molecules (distances shorter than sum of vdW radii). The different locations of the close contacts originate from the differences in the main intermolecular interactions occurred in the two polymorphic forms. The interactions between the selected atom types are calculated separately and visualized on the Hirshfeld surfaces. The comparison of the main O…H and C…H interactions is shown in Figures S2 and S3.

**Figure 6:**
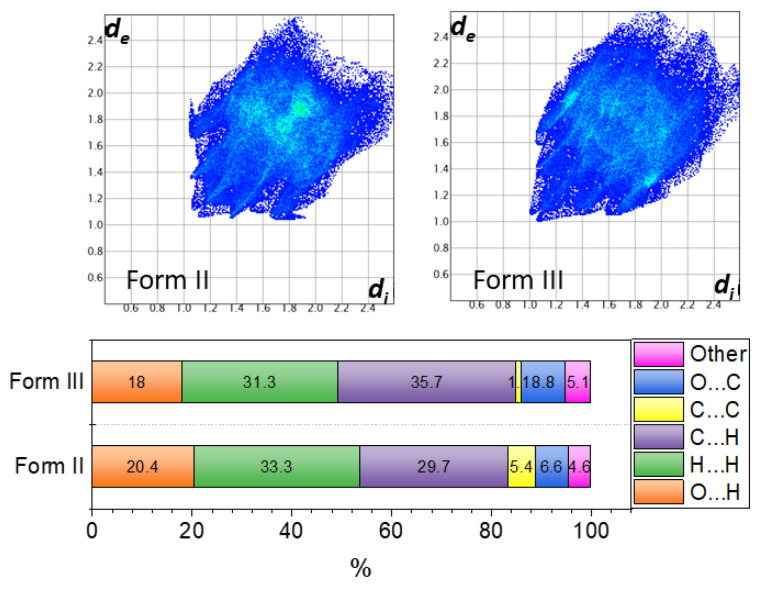
Comparison of the fingerprint plots of Forms II and III (**upper part**) and the relative contribution of the various intermolecular contacts to the Hirshfeld surface area (**lower part**).

The 2D fingerprint plots have been calculated to investigate the contributions of different secondary interactions that are responsible for the different crystal packings observed in the two crystals. These plots change highly sensitively to the environment of the molecule, and are unique for a given molecular conformation even in its particular polymorphic forms. Figure 6 shows the 2D fingerprint plots calculated for Forms II and III and the percentage contributions of the various close intermolecular atomic contacts to the Hirshfeld surface area. Though the 2D fingerprint plots are very similar in the two forms, there are some significant differences in the relative contributions of a few intermolecular contacts to the Hirshfeld surface area (see also Figure S4). Comparing the two forms, significantly fewer (2.4%) O…H contact contributions could be found in Form III than in Form II. In case of Form II, five neighboring molecules were found to have relatively short O…H interactions with the selected molecule, while in Form III only four did (see Figure S3). At the same time the number of C…H contacts is 6.0% higher in Form III compared to Form II, which can be due to the packing arrangements where the aromatic rings are positioned edge-to-face or parallel off-stacking (see Figure S4). This also explains the decrease in C…C interactions in Form III (1.1%), where the face-to-face arrangements of the aromatic rings are missing. 

### 2.4. Interaction Energy and Energy Frameworks

**Figure 7:**
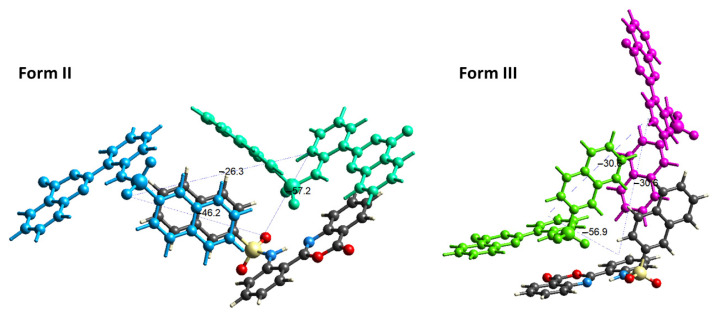
Color-coded interaction mapping within 3.8 Å of the reference molecule for polymorphic crystals Form II and Form III with the obtained E_tot_ values between selected molecular pairs (full data are collected in Table S1).

As the polymorphs Form II and Form III contain two conformations of the same molecule, this gave us the opportunity to examine how the difference in conformation only and exclusively affects the arrangement of the molecules. First, the electrostatic potential was calculated and mapped over the Hirshfeld surface to compare the differences in the electron distribution. Then, the calculation of pair-wise interaction energies within a crystal by summing up four energy components comprising electrostatic (E_ele_), polarization (E_pol_), dispersion (E_dis_) and repulsion (E_rep_) was performed using the B3LYP/6-31G(d,p) energy model. For this energy calculation, molecules within 3.8 Å of a reference molecule have been taken into account. The obtained pair-wise interaction energies with color codes are collected in Table S1. Figure 7 shows the molecule selected for the calculation (colored by element) and the position of the two neighboring molecules with the lowest interaction energies. It can be seen that in Form II, two molecules turn to each other with their naphthalene rings (DE) parallel, while this ring–ring interaction is missing in Form III due to the interposition of a third (magenta) molecule. All calculated interaction energies and color codes are collected in Table S1.

**Figure 8:**
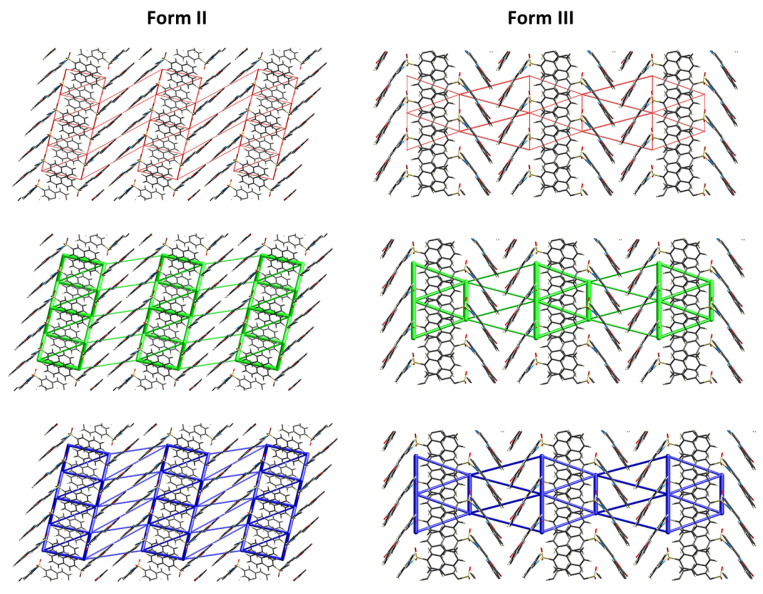
Energy frameworks calculated for a cluster of 3 × 3 × 3-unit cells for polymorphs Form II and Form III viewed along the *b*-axis for Form II and along the *a*-axis for Form III, showing the (**top**) electrostatic potential force, (**middle**) dispersion force and (**bottom**) total energy diagrams. (The cylindrical radii are proportional to the relative strength of the corresponding energies, and these were adjusted to the same scale factor of 60 with a cut-off value of 9 kJ/mol.).

Figure 8 compares the shape of the electrostatic potential force, dispersion force and total energy frameworks calculated for their clusters of 3 × 3 × 3-unit cells. The importance of this functionality is pronounced in the case of polymorphs, as it allows users to directly compare the topological differences in the energy components between the structures, which may be correlated with physicochemical properties. In our case, the topology of the total energy is significantly governed by electrostatic forces, for which the directionality differs considerably due to the different molecular arrangements in the two crystals. The packing arrangements are viewed along the b-axis for Form II and along the a-axis for Form III, which clearly shows the difference between the two forms, i.e., the difference in the orientation of the molecular columns caused by the conformation of the molecules.

### 2.5. Characterization of Polymorphic Modifications by Powder XRD, DSC, and FTIR Spectroscopy

**Figure 9:**
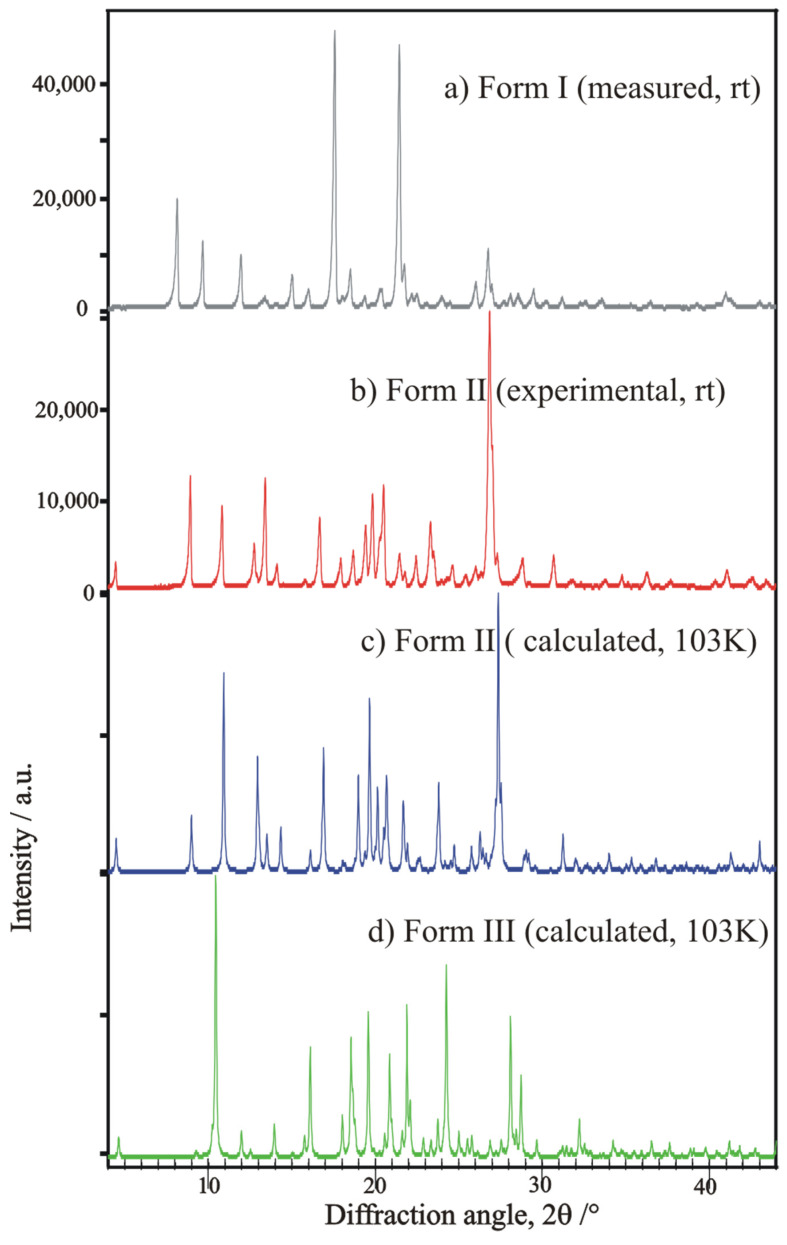
Comparison of powder XRD-patterns of the three polymorphic forms of *N*-(2-(4-oxo-4*H*-benzo[d]1,3oxazin-2-yl)phenyl)naphthalene-2-sulfonamide: (**a**,**b**) experimentally measured the pattern of Sample **1** (Form I) and Sample **2** (Form II), respectively, at room temperature; (**c**,**d**) calculated patterns based on our single-crystal structure determination of Form II and III, respectively, determined at 103 K.

The measured experimental powder XRD patterns of ‘Sample 1 (Forms I)’ and ‘Sample 2 (Form II) of *N*-(2-(4-oxo-4*H*-benzo[d]1,3oxazin-2-yl)phenyl)naphthalene-2-sulfonamide are shown in Figure 9 in comparison with the calculated ones from our single-crystal structure determinations. The crystallographic unit cell information obtained by single-crystal determinations in comparison with our estimations obtained from powder XRD patterns using the DASH program package are summarized in Table 5.

**Figure 10:**
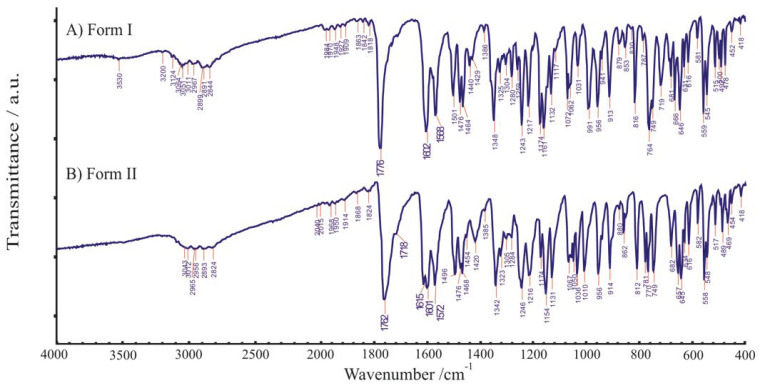
FTIR spectra of Sample **1** (Form I, **A**) and Sample **2** (Form II, **B**). The two forms differ significantly in the wavenumber of carbonyl stretching vibrations, namely 1776 and 1762 cm^−1^, respectively. The value of the carbonyl stretching vibration in Form III, for comparison, is 1764 cm^−1^ [2].

The FTIR spectra of Sample 1 (Form I) and Sample 2 (Form II) differ significantly in all regions, mainly in the wavenumbers of carbonyl stretching vibrations, such as 1776 and 1762 cm^−1^ for Form I and II, respectively (Figure 10). (Note: These bands can be compared with the limited data provided for Form III (1764 cm^−1^) [2]). Anyhow, no sharp individual νNH stretching vibrations are observed (as it would have been expected), probably because of the very strong N-H … N hydrogen bonds (observed by the single-crystal structural determinations), which seem to be evenly distributed in a very wide range of wavenumbers between 3300 and 2000 cm^−1^ in both cases, mostly as baseline shifts.

**Figure 11:**
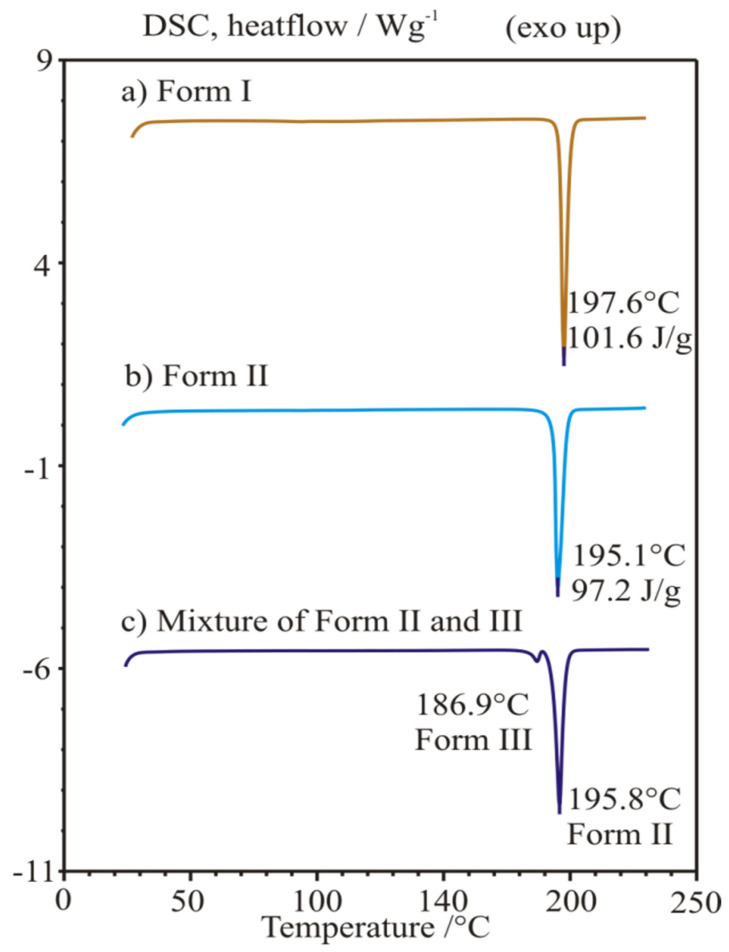
DSC melting curves of (**a**) Sample 1 (Form I), (**b**) Sample 2 (Form II), and (**c**) Sample 3, (**a**) crystal mixture of Form II and III recrystallized from Form I of *N*-(2-(4-oxo-4*H*-benzo[d]1,3-oxazin-2-yl)phenyl)naphthalene-2-sulfonamide. Form I has the highest melting point of 197–198 °C, while Form II fuses at about 195–196 °C and Form III at 186–187 °C (lit. 184.8–185.5 °C [2]).

DSC melting curves of Sample 1 (Form I) and Sample 2 (Form II), and Sample 3 (a mixture of Form II and III) are presented in Figure 11. Form I has the highest melting point of 197–198 °C, while Form II fuses at about 195–196 °C and Form III at about 186–187 °C (lit. 184.8–185.5 °C [2]. Unfortunately, pure Form III has not been available for repeated DSC study. Based on the published melting point and IR data noted by Bratscley et al. [21], it might be assumed that Form II-type crystalline matter was achieved already in 1988.

### 2.6. Comparison of Solid-State Fluorescence of the Polymorphic Forms of Sample 1 (Form I, “UV-Yellowish”) and Sample 2 (Form II, “UV-Greenish”)

**Figure 12:**
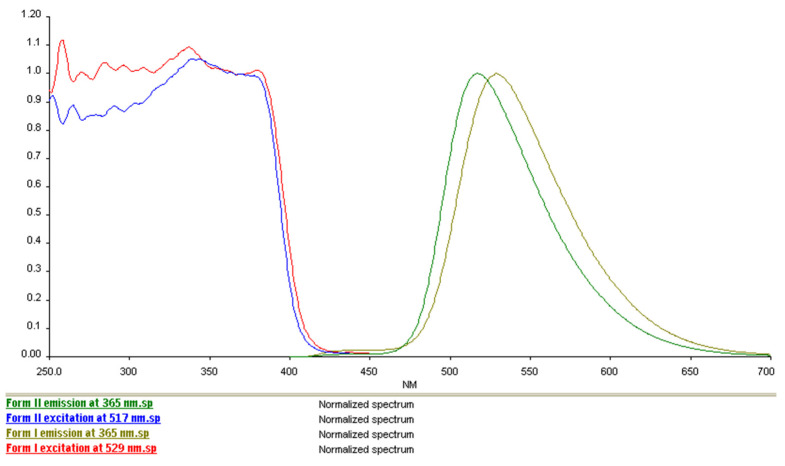
Normalized excitation (blue and red curves) and emission (green and yellowish green curves) spectra of the Form II and Form I samples, respectively. (UV emission spectra of the new solid modifications were measured at λ = 365 nm, while their excitation spectra are obtained at wavelengths of their emission maximum, at λ_max_= 517 and 529 nm for Form II (more greenish) and I (more yellowish), respectively).

UV emission spectra of the two new solid modifications (Forms I and II) measured at λ = 365 nm; their excitation spectra obtained at wavelengths of their emission maximum, at λ_max_ = 517 and 529 nm for Form II (more greenish) and I (more yellowish), respectively, are shown in Figure 12.

### 2.7. Simultaneous Thermogravimetric and Differential Thermal Analysis (TG/DTA) Coupled with Quadrupole Mass Spectroscopy for Evolved Gas Analysis of the Polymorphic Forms Sample 2 (Form II, “UV-Greenish”) Prepared in Dichloromethane

**Figure 13:**
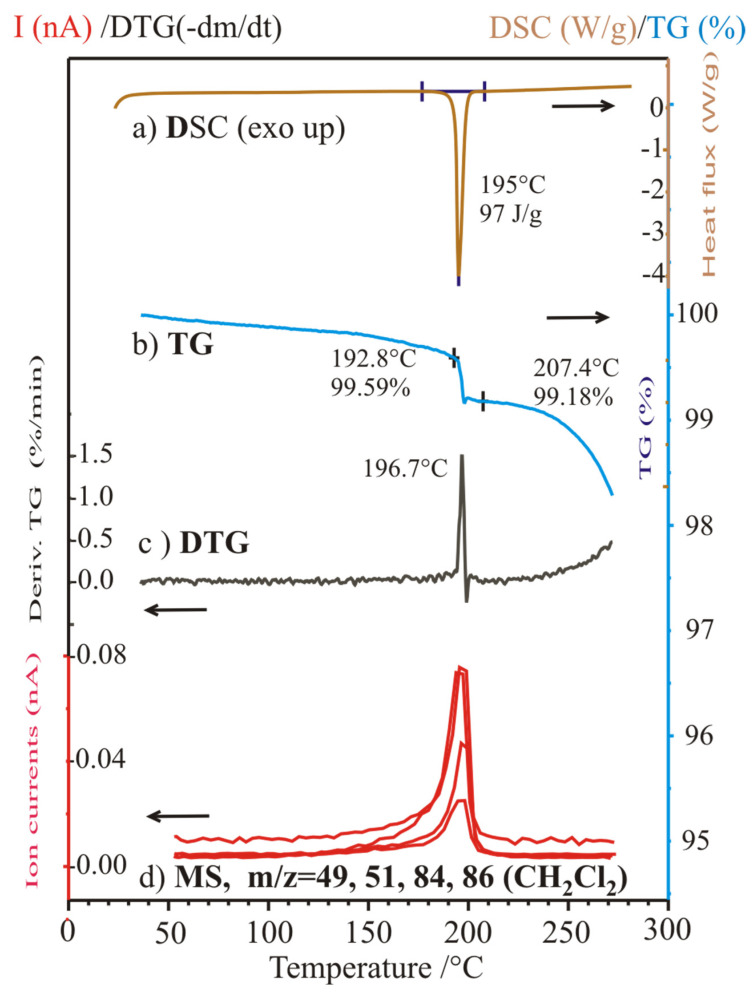
Curves of simultaneous thermogravimetric and differential thermal analysis (TG/DTA) coupled with quadrupole mass spectroscopy for evolved gas analysis of the polymorphic forms of Sample 2 (Form II, “UV-greenish”) prepared in dichloromethane. (**a**) The DSC melting curve shows the heat flow of fusion process (top, brown, actually replacing the raw DTA curve; (**b**) thermogravimetric TG curve (second from the top) exhibits enlarged a minor weight loss during the fusion; (**c**) time-derivative DTG curve (second from the bottom) reflects the rate dynamics of this minor weight loss escorting the melting procedure; (**d**) specific ion current changes (bottom) observed by MS for m/z = 49, 51, 84, and 86 (mass/charge, amu), representing the release of a small amount of residual solvent dichloromethane, CH_2_Cl_2_, impurity during the fusion.

Checking for minor contaminations and solvent residues originating from the synthetic processes, curves of simultaneous thermogravimetric and differential thermal analysis (TG/DTA) coupled with quadrupole mass spectroscopy of the polymorphic forms Sample 2 (Form II, “UV-greenish”) prepared in dichloromethane, shows an unexpected gas evolution during its melting process (Figure 13). The thermogravimetric (TG) curve (Figure 13b, second from the top) exhibits a minor weight loss during the fusion, which was seen to be enlarged by the time-derivative (DTG) curve (Figure 13c) reflecting the rate dynamics of this minor weight loss escorting the melting procedure. Meanwhile, some specific ion current changes (bottom) also occur as observed by MS for m/z = 49, 51, 84, and 86 (mass/charge, amu), representing the release of a small amount of inclusion solvent, CH_2_Cl_2_, during the fusion of Sample 2 (Form II, “UV-greenish”) shown in Figure 13d). The evolution of dichloromethane likely remained in the crystals as the solvents’ inclusions. Similar gas evolutions cannot be observed for Sample 1 (Form I, “UV-yellowish”).

## 3. Materials and Methods

### 3.1. X-Ray Data Collection, Structure Solution and Refinement

Colorless platelet crystals of Form II and block shape crystals of Form III were measured at –170 °C on a Rigaku RAXIS-RAPID II diffractometer (Akishima, Japan) using Mo-Kα radiation. Numerical absorption corrections [25] were carried out using the program CrystalClear-SM 1.4.0 SP1 [26].

Sir2014 [27] and SHELXL [28] under WinGX [29] software were used for structure solution and refinement, respectively. The models were refined anisotropically on a full-matrix by the least squares method on F^2^. A summary of the crystal data, data collection, structure determination and refinement parameters is given in Table 2. The refinement of non-hydrogen atoms was carried out with anisotropic displacement parameters. The hydrogen atomic positions were located in different electron density maps, then constrained into geometric positions. They were included in structure factor calculations, but they were not refined. The isotropic displacement parameters of the hydrogen atoms were approximated from the U(eq) value of the atom they were bonded to. Selected bond lengths and angles of the compounds were calculated by PLATON software [30]. The graphical representation and the edition of CIF files were performed by Mercury [31] and PublCif [32] software, respectively.

### 3.2. Hirshfeld Surface Analysis and Energy Calculations

The Hirshfeld surfaces of the investigated molecules in Form II and III were calculated by Crystal Explorer 17.5. [33,34,35] using the function d_norm_ (normalized contact distance). The Hirshfeld surface of a molecule is generated by points where the contribution to the electron density from the molecule of interest is equal to the contribution from all neighboring molecules. Each point of this surface has two distances: d_e_, the distance from the point to the nearest nucleus external to the surface, and d_i_, the distance to the nearest nucleus internal to the surface. The combination of d_e_ and d_i_ in the form of a 2D fingerprint plot results in a unique property of each crystal and provides a useful tool to compare the intermolecular contacts in different crystals. In the Hirshfeld surface calculations, all bond lengths of C-H, O-H and N-H were set to the normalized values defined by neutron diffraction (1.083 Å, 0.983 Å and 1.009 Å, respectively).

The interaction energies were calculated for a cluster which was built up from molecules within a sphere of a 3.8 Å radius of the reference molecule using the B3LYP/6-31G(d,p) quantum level of theory available in Crystal Explorer 17.5. The total intermolecular energy consists of the following energy components: electrostatic, dispersion, polarization and repulsion. These were summed up with the scale factors of 1.057, 0.871, 0.740 and 0.618, respectively [33].

### 3.3. Further Analytical Methods (CHN, FTIR, NMR, DSC, TG/DTA-EGA-MS, XRD, UV)

The CHN-elemental analyses of new ‘Sample 1 (Form I)’ and ‘Sample 2 (Form II)’ were carried out using a Vario EL III (Elementar Analysensysteme GmbH, Langenselbold, Germany) CHN-O Analyser, while their sulfur content was determined by the method provided by Shöniger [36]. No pure bulk sample containing Form III was available for studying.

The FT-IR spectra of Samples **1**–**4** were measured by an Excalibur Series FTS 3000 (Bio-Rad, Hercules, CA, USA) FTIR spectrophotometer in KBr between 400 and 4000 cm^−1^, while their ^1^H and ^13^C-NMR spectra were recorded on a Bruker Advance 300 MHz NMR spectrometer (Bruker, Billerica, MA, USA). In the latter cases, samples were dissolved in CDCl_3_, and the chemical shifts were measured to TMS, at 24 °C. The control software was XWinNMR 3.5.

Differential scanning calorimetry (DSC) measurements were performed using a Modulated DSC 2920 device (TA Instruments, New Castle, DE, USA). The samples (1–5 mg) were measured in sealed aluminum pans at a heating rate of 10 K/min. For temperature and enthalpy calibration of the DSC instrument, pure metal standard was applied.

A simultaneous thermogravimetric and differential thermal analysis (TG/DTA) apparatus (STD 2960 Simultaneous DTA-TGA, TA Instruments Inc., USA, DE, Delawar), a heating rate of 10 °C/min, an air flow rate of 130 mL min^−1^, and sample sizes between 10 and 55 mg were applied; this analysis was carried out in an open Pt crucible. The mixture of the gaseous species was examined using the ThermoStar GDS 200 (Balzers Instruments, Balzers, Liechtenstein) quadrupole mass spectrometer equipped with a Chaneltron detector (Surface Concept GmbH, Mainz, Germany), through a heated 100% methyl deactivated fused silica capillary tubing that was kept at T = 200 °C. Data collection was carried out using Quad-Star 422v7.02 software in Multiple Ion Detection (MID) mode, monitoring only 64 selected channels based on changes observed in scanning mode. The measuring time was ca. 0.5 s for one channel, resulting in a measuring time of ca. 38 s for each MID cycle.

Powder XRD patterns were recorded with an X’pert Pro MDP (PANalytical Bv., Almelo, The Netherlands) X-ray diffractometer equipped with an X’celerator detector using Cu Kα radiation and a Ni filter.

The DASH software package [37] was used [24] for indexing the powder pattern of Sample 1 (Form I) and 2 (Form II) at room temperature, and for space group determination from a statistical assessment of systematic absences [38].

UV emission spectra of the new solid modifications were measured at λ = 365 nm, while their excitation spectra were obtained at wavelengths of their emission maximum, at λmax = 517 and 529 nm for Form II (more greenish) and I (more yellowish), respectively, using the Perkin Elmer LS 55b spectrofluorimeter (Waltham, MA, USA). The powder samples were measured as spread on whitening-free paper placed into a home-made solid sample holder, with an accumulation number of 10, and some further parameters (cut-off filter 390 nm, exslit 10, emslit 0, and Hamamatsu R955-type PM).

## 4. Conclusions

It is known that the phenomenon of polymorphism occurs relatively frequently among sulfonamide-type organic compounds. In particular, a lot of examples can be found among active pharmaceutical ingredients. Two of the most famous cases are of Sulfapyridine [39,40,41,42,43,44] and Sulfathiazole [39,45,46,47,48]. Among 2-(2′-arylsulfonylaminophenyl)-4*H*-3,1-benzoxazin-4-one-type fluorescent dyes, we have firstly observed two new polymorphic crystalline modifications (Forms I and II) with significantly different melting points of 195–196 and 197–198 °C, respectively, for the naphthyl derivative, beside the previously known Form III with a melting point of 184.5–185.5 °C as recorded in the literature. Both the ^1^H and ^13^C NMR spectra obtained in CDCl_3_ of the new modifications show the same chemical shifts, which correspond to their identical chemical formulae.

Powder XRD patterns of Forms I and II have been found to differ from each other and from that of generated on base of the single-crystal atomic coordinates of Form III, either obtained in this work or for structure YOCTAO [6]. Single crystals could be grown from Form II; its structure has been determined (s.g. No. 4, *P*2_1_/*c*) with lattice parameters as follows: a = 20.2155(10), b = 8.8822(4), c = 11.0016(4) Å, β = 103.6160(10) ° and V = 1919.91(15) Å^3^). Instead of Form I, a single crystal of Form III could be harvested during its recrystallization trials from any appropriate solvents, including glacial acetic acid, as well. The re-determined structure measured at T = 103 K agrees with the former structure obtained at T = 173 K [6]. The FT-IR spectra of polymorphs have also been found to be significantly different from each other, and none of them show any significant νNH stretching vibrations as the sulfonamidic -NH groups are involved in very strong hydrogen N-H…N bonds (D…A distances are ca. 2.65 Å in both cases). The fluorescent emission spectra of the two new modifications are also significantly different, with emission maximum at wavelengths at λ_max_ = 517 and 529 nm for Form II (more greenish) and I (more yellowish), respectively. Unfortunately, the pure bulk sample of Form III with reported λ_max_ = 520 nm [6] can no longer be obtained.

Efforts have been made toward revealing reproducible conditions under which polymorphs are formed. Tt seems that the solvents (see, e.g., the solvatomorphic hemi-p-xylene crystal of YOCSUH) or other probable organic contaminations are most likely to be responsible for the formation, nucleation and growth of crystals of various polymorphic forms. The release of dichloromethane as a residual solvent impurity from Sample 2 (Form II) has been detected using TG-EGA-MS methods.

To uncover the similarities and differences in their packing arrangement and their secondary interaction systems, all of which affect the molecular conformations in their crystals, lattice energy calculations have also been carried out beyond performing detailed Hirshfeld surface analyses.

## Figures and Tables

**Table 1:** Reported melting points and/or single-crystal structures of 2-(2′-arylsulfonylaminophenyl)-4*H*-3,1-benzoxazin-4-one derivatives ^1^.

Compounds, CAS Reg. No., mp(s), [Ref(s)]	Compounds, CSD Code(s), [Ref(s)], SG
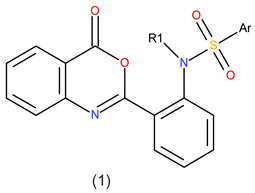	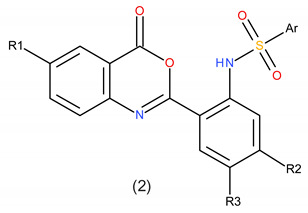
**1a, Ar = Ph, R1 = H** (CAS 10128-51-5, 214–215 °C [2], 218–219 °C [7], 219–220 °C [8])	**2a, R1 = R2 = R3 = H, Ar = 4-MePh** (TAPBZO) [5], *P*-1
**1b, Ar = 4-MePh, R1 = H** (CAS 3808-20-6, 220–220.8 °C [2], 219 °C [9], 221 °C [10], 221 °C [11])	**2b, R1 = Br, R2 = R3 = H, Ar = 4-MePh** (BEHHOL [12], BEHHOL10 [13]), *P*2_1_/*a*
**1c, Ar = β-naphthyl, R1 = H** (CAS 10128-55-9, 184.8–185.5 °C [2])	**2c, R1 = R3 = H, R2 = OCH_3_, Ar = 4-MePh** (FEYJEY and FEYJEY01 [13]), *P*2_1_/*b*
**1c, Ar = α(β?)-naphthyl, R1 = H** (CAS 3779-52-0, 184.8–185.5 °C [11])	**2d, R1 = R2 = H, R3 = NO_2_, Ar = 4-MePh** (TSBZON [14], TSBZON01 [15]), *Pbn*2_1_
**1e, Ar = 2,4,6-Me_3_Ph, R1 = H** (CAS 3780-70-9, 223–223.5 °C [2] 224 °C [11])	**2e, R1 = R3 = H, R2 = NO_2_, Ar = 4-MePh** (TSBZOX [16]), *P*-1
**1f, Ar = 2-MePh, R1 = H** (CAS 10128-56-0, 222–223 °C [2])	**2f, R1 = R2 = R3 = H, Ar = β-naphthyl** (YOCTAO [6]), *P*2_1_
**1g, Ar = 4-iPrPh, R1 = H** (CAS 3808-65-9, 185 °C [11])	**2g, R1 = R2 = R3 = H, Ar = β-naphthyl, 0.5 p-xylene solvate** (YOCSUH [6]), *P*-1
**1h, Ar = 4-NO_2_Ph, R1 = H** (CAS 10128-57-1, 225.5–226.5 °C [2])	
**1i, Ar = 4-MePh, R1 = CH_3_** (CAS 10129-14-3, 176–177 °C [2])	

^1^ **1b** and **2a**, **1c** and **2f** are pairwise identical compounds, indicated by the same background colors.

**Table 2:** ^13^C NMR signals of Sample **1** (Form I) and Sample **2** (Form II), in comparison (relative to TMS).

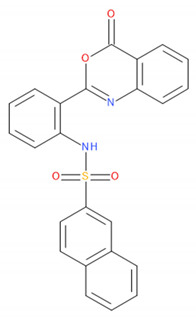	**13C Chemical Shifts** **Peak No.**	**Sample 1** **δ (ppm)**	**Sample 2** **δ (ppm)**
	1	157.7	157.7
	2	157.0	157.0
	3	144.8	144.7
	4	139.5	139.5
	5	137.0	137.0
	6	136.2	136.2
	7	134.8	134.8
	8	134.0	133.9
	9	131.8	131.8
	10	129.6	129.6
	11	129.4	129.4
	12–13, not resolved	129.1	129.1
	14	128.9	128.9
	15	128.8	128.7
	16	128.7	128.7
	17	127.8	127.8
	18	127.5	127.5
	19	126.5	126.5
	20	123.3	123.3
	21	121.9	121.9
	22	119.4	119.4
	23	116.5	116.5
	24	115.4	115.4

**Table 3:** Crystal data and details of data collection, structure determination and refinement for Forms II and III.

	Form II	Form III
Empirical formula	C_24_H_16_N_2_O_4_S	C_24_H_16_N_2_O_4_S
Formula weight (g/mol)	428.45	428.45
Crystal color and shape	Colorless, Platelet	Colorless, Block
Temperature (K)	103(2)	103(2)
Radiation and wavelength (Å)	Mo-Kα, 0.71073	Mo-Kα, 0.71073
Crystal system	monoclinic	monoclinic
Space group	*P*2_1_/*c*	*P*2_1_
Unit cell dimensions ^a^		
a (Å)	20.2155(10)	8.7804(7)
b (Å)	8.8822(4)	5.7461(6)
c (Å)	11.0016(4)	19.3481(19)
β (°)	103.6160(10)	100.425(2)
Volume (Å^3^)	1919.91(15)	960.06(16)
*Z/Z’*	4/1	2/1
Density (calc.) (mg m^−3^)	1.482	1.482
Absorption coefficient, μ (mm^−1^)	0.206	0.206
*F*(000)	888	444
Crystal size (mm)	0.50 × 0.30 × 0.10	0.50 × 0.12 × 0.12
Absorption correction	Numerical	Numerical
Max. and min. transmission	0.9297, 0.9774	0.9543, 0.9781
θ?range for data collection (°)	3.006 ≤ θ ≤ 27.473	3.212 ≤ θ ≤ 25.350
Index ranges	−26 ≤ *h* ≤ 26; −11 ≤ *k* ≤ 11; −14 ≤ *l* ≤ 14	−10 ≤ *h* ≤ 10; −6 ≤ *k* ≤ 6; −23 ≤ *l* ≤ 23
Reflections collected	42568	12343
Completeness to 2θ	0.999	0.997
Independent reflections (R_int_)	4392, 0.0645	3510, 0.0924
Reflections *I* > 2σ(*I*)	3339	2514
Refinement method	full-matrix least-squares on *F*^2^	full-matrix least-squares on *F*^2^
Data/restraints/parameters	4392/0/280	3510/0/280
Goodness-of-fit ^b^, on *F*^2^	1.095	1.048
Final *R* indices ^c,d^ [*I* > 2σ(*I*)] (*R*1, *wR*2)	0.0425, 0.1016	0.0551, 0.1069
R indices ^c,d^ (all data) (*R*1, *wR*2)	0.0589, 0.1098	0.0881, 0.1249
Max. and mean shift/esd	0.000; 0.000	0.000; 0.000
Largest diff. peak and hole (e.Å^−3^)	0.370; −0.304	0.340; −0.347

^a^ Uncertainties (SD) of the last digits are shown in parentheses. ^b^ GOF = {∑[*w*(*F*_o_^2^ − *F*_c_^2^)^2^]/(*n* − *p*)}^1/2^, where *n* is the number of reflections and *p* is the total number of parameters refined. ^c^ R_1_ = ∑∥F_o_∣ − ∣F_c_∥/∑∣F_o_∣; ^d^ wR_2_ = {∑[*w*(F_o_^2^ − F_c_^2^)^2^]/∑[*w*(F_o_^2^)^2^]}^1/2^.

**Table 4:** Selected intra- and intermolecular interactions in Forms II and III of *N*-(2-(4-oxo-4*H*-benzo[d]1,3oxazin-2-yl)phenyl)naphthalene-2-sulfonamide.

*D*—X…*A*	*D*—X (Å)	X…*A* (Å)	*D*…*A* (Å)	*D*—X…*A* (°)	Symmetry Code
**Form II**					
N2-H2N…N1	0.88	1.94	2.654(2)	137	intra
C11-H11…O3	0.95	2.31	2.988(2)	128	intra
C14-H14…O1	0.95	2.66	2.295(2)	102	intra
C16-H16…O3	0.95	2.91	2.554(2)	103	intra
C4-H4…O2	0.95	2.51	3.270(2)	138	−x, −1/2 + y, 3/2 − z
C12-H12…O4	0.95	2.51	3.130(2)	123	x, 1 + y, z
C13-H13…O4	0.95	2.61	3.168(2)	118	x, 1 + y, z
C24-H24…O3	0.95	2.54	3.249(2)	132	x, 3/2 − y, 1/2 + z
C18-H18…Cg(D) ^a^	0.95	2.84	3.6457(19)	143	1 − x, 1/2 + y, 1/2 − z
C20-H20…Cg(C) ^a^	0.95	2.58	3.512(2)	167	1 − x, 2 − y, 1 − z
S1-O4…Cg(A) ^a^	1.4317(12)	3.0870(14)	3.8940(8)	113.92(6)	x, 3/2 − y, −1/2 + z
**Form III**					
N2-H2N…N1	0.88	1.95	2.665(7)	137	intra
C11-H11…O3	0.95	2.45	3.092(7)	125	intra
C14-H14…O1	0.95	2.67	2.309(7)	102	intra
C16-H16…O3	0.95	2.84	2.438(7)	105	intra
C3-H3…O2	0.95	2.62	3.564(7)	171	1 − x, −1/2 + y,1 − z
C12-H12…O4	0.95	2.52	3.105(6)	120	1 + x, y, z
C13-H13…O4	0.95	2.56	3.133(7)	119	1 + x, y, z
C21-H21…O4	0.95	2.64	3.485(7)	149	−x, −1/2 + y, − z
C4-H4…Cg(B) ^a^	0.95	2.97	3.819(6)	149	−x, −1/2 + y, 1 − z
C18-H18…Cg(E) ^a^	0.95	2.77	3.514(6)	135	1−x, 1/2 + y, − z
C23-H23…Cg(D) ^a^	0.95	2.83	3.551(6)	134	−x, −1/2 + y, − z
C1-O2…Cg(A) ^a^	1.196(7)	3.050(4)	4.063(6)	142.3(4)	1−x, −1/2 + y, 1 − z

^a^ Cg is the centre of gravity of the indicated ring.

**Table 5:** Unit cell parameters of the various polymorphic forms of *N*-(2-(4-oxo-4*H*-benzo[d]1,3oxazin-2-yl)phenyl)naphthalene-2-sulfonamide obtained by single-crystal structure determinations in comparison with those of estimations from the measured powder XRD patterns by indexing [23] using the interactive DASH program [24].

Unit Cell Parameters	Form I, Powder Pattern Indexing at RT, by DASH [This Work]	Form II, Powder Pattern Indexing at RT, by DASH [This Work]	Form II Single-Crystal Structure Determination at T = 103 K [This Work]	Form III Single-Crystal Structure Determination at T = 103 K [This Work]	Form III Single-Crystal Structure Determination at T = 173 K [6]
space group	*P* 2_1_/*a* *No. 14*	*P* 2_1_/*c* *No. 14*	*P* 2_1_/*c* *No. 14*	*P* 2_1_ *No. 4*	*P* 2_1_ *No. 4*
*a* (Å)	21.945	20.269	20.2155(10)	8.7804(7)	8.778(4)
*b* (Å)	10.128	8.9526	8.8822(4)	5.7461(6)	5.771(3)
*c* (Å)	9.0333	11.205	11.0016(4)	19.3481(19)	19.308(9)
*β* (°)	95.461	102.84	103.6160(10)	100.425(2)	100.446(7)
V (Å^3^)	1998.7	1982.5	1919.91(15)	960.06(16)	962.0(8)
Z/Z′	4/1	4/1	4/1	2/1	2/1
zero shift (°)	0.0798	0.0145	-	-	-

RT = room temperature.

## Data Availability

CCDC 2418661-2418662 contain the supplementary crystallographic data of Forms II and III of *N*-(2-(4-oxo-4*H*-benzo[d]1,3oxazin-2-yl)phenyl)naphthalene- 2-sulfonamide, respectively. These data can be obtained free of charge from The Cambridge Crystallographic Data Centre via www.ccdc.cam.ac.uk/data_request/cif (accessed on 20 March 2025).

## Supplementary Materials

The following supporting information can be downloaded at: https://www.mdpi.com/article/10.3390/molecules30081671/s1, Figure S1: Packing arrangements of the molecules; Figure S2: Comparison of the O…H interactions in Form II and III showing the Hirshfeld surfaces. Neighboring molecules associated with close contacts are shown.; Figure S3: Comparison of the C…H interactions in Form II and III showing the Hirshfeld surfaces. Neighboring molecules associated with close contacts are shown.; Figure S4: Contributions of specific pairs of atom types to the full 2D fingerprint plots calculated in the crystals of Form II and Form III. Figure S5. Colour-coded interaction mapping within 3.8 of the reference molecule for polymorphic crystals Form II and Form III with the obtained E_tot_ values between selected molecular pairs. Table S1. Color-coded interaction energies calculated for polymorphic crystals of Form II and Form III.

## References

[1] CAS SciFinder(n) Literature and Chemical Compound Database. https://scifinder-n.cas.org/search/substance/66bb509d5d94650a0109de8f/1.

[2] Bolotin B.M., Ryabokobylko Y.S., Drapkina D.A., Brudz V.G. (1965). Synthesis and optical properties of 2-phenyl-4H-3,1-benzoxazin-4-one and its derivatives. Tr. Vses. Nauchn.-Issled. Inst. Khim. Reakt. I Osob. Chist. Khim. Veshchestv.

[3] Groom C.R., Bruno I.J., Lightfoot M.P., Ward S.C. (2016). The Cambridge structural database. Struct. Sci..

[4] Cambridge Crystallographic Data Centre (CCDC) Crystal Structure Database (CSD), Release v.5.45, + 2 Updates. https://www.ccdc.cam.ac.uk/solutions/csd/.

[5] Filipenko O.S., Ponomarev V.I., Bolotin B.M., Atovmyan L.O. (1979). Crystal and molecular structure of a new organic luminophor 2-(2-tosylaminophenyl)-4H-3,1-benzoxazin-4-one. Crystallogr. Rep..

[6] Bolotin B.M., Mikhlina Y.A., Arkhipova S.A., Kuz’mina L.G. (2012). X-ray diffraction study of crystals of the organic luminophore Orlyum White 520T. Crystallogr. Rep..

[7] Bolotin B.M., Drapkina D.A., Brudz V.G., Sudiyarova R.U. (1968). 2-Aryl-4H-3,1-benzoxazin-4-ones and their analogs. II. Some reactions of o-acylaminobenzamides In Zhurnal Vsesoyuznogo Khimicheskogo Obshchestva im. D. I. Mendeleeva.

[8] Wakankar D.M., Hosangadi B.D. (1978). Studies in large ring compounds: Part IV. Reactions of N-substituted anthranilic acids with polyphosphate ester and dicyclohexylcarbodiimide. Indian J. Chem. Sect. B Org. Chem. Incl. Med. Chem..

[9] Davis M., Hook R.J., Wu W.Y. (1984). 2,1-Benzisothiazoles. XIII. Intermediates and products in the sulfur extrusion reaction of 2,1-benzisothiazolin-3(1H)-one. A new synthesis of 11H-pyrido[2,1-b]quinazolin-11-one (“pyracridone”). J. Heterocycl. Chem..

[10] Khimich M.N., Birgen E.A., Bolotin B.M., Uzhinov B.M. (2009). Intramolecular photoinduced proton transfer in 2-(2-aminophenyl)-4H-3,1-benzoxazin-4-ones with different electron-withdrawing N-substituents. High Energy Chem..

[11] (1965). All-Union Scientific-Research Institute of Chemical Reagents and Pure Chemical Substances. 2-(2-Arylsulfonamidophenyl)-4H-3,1-Benzoxazin-4-Ones. French Patent application.

[12] Filipenko O.S., Atovmyan L.O., Ponomarev V.I., Bolotin B.M. (1981). Molecular and crystal structure of the organic luminophor 2-(2-tosylaminophenyl)-6-bromo-4H-3,1-benzoxazin-4-one. Bull. Acad. Sci. USSR Div. Chem. Sci..

[13] Filipenko O.S., Ponomarev V.I., Atovmyan L.O., Bolotin B.M. (1987). Molecular and crystal structure of organic phosphors 2-(2′-tosylaminophenyl)-6-bromo-4H-3,1-benzoxazin-4-one at 300 K and 2-(2′-tosylamino-4-methoxyphenyl)-4H-3,1-benzoxazin-4-one at 300 and 100 K. Crystallogr. Rep..

[14] Filipenko O.S., Bolotin B.M., Atovmyan L.O., Ponomarev V.I. (1980). Molecular and crystal structure of the organic luminophor 2-(2-tosylamino-5-nitrophenyl)-4H-3,1-benzoxazin-4-one. Sov. Phys. Dokl..

[15] Utenyshev A.N., Filipenko O.S., Bolotin B.M., Ponomarev V.I. (2001). Low-temperature study of the molecular and crystal structures of 2-(2′-tosylamino-5′-nitrophenyl)-4H-3,1-benzoxazin-4-one, an organic luminophor. Crystallogr. Rep..

[16] Filipenko O.S., Atovmyan L.O., Bolotin B.M., Ponomarev V.I. (1981). Crystal and molecular structure of the organic luminophor 2-(2-tosylamino-4-nitrophenyl)-4H-3,1-benzoxazin-4-one. Crystallogr. Rep..

[17] Bolotin B.M., Kurnosova L.S., Koroljkova O.N., Drapkina D.A., Terskoi J.A., Sinjaver L.G., Brudz V.G., Jarovaja G.D. (1967). Process of Manufacturing Substitued Benzoxazin-4-Ones. Patent Specification.

[18] Bolotin B.M., Kurnosova L.S., Koroljkova O.N., Drapkina D.A., Terskoi J.A., Sinjaver L.G., Brudz V.G., Jarovaja G.D. (1967). Process of Manufacturing Substitutied 2(21-Arylsulphonyl Aminophenyl)-4H,3,1-Benzoxazin-4-Ones.

[19] Cevasco A.A. (1972). Substituierte Sulfonamido-Aund Carboxamidophenylchazolone. German (BRD) Patent Application Offenlegegungsschrift.

[20] Cevasco A.A. (1973). Certain 2-(o-Sulfonamidophenyl)-4(3H)-Quinazolineones. U.S. Patent Application.

[21] Bratscley R., Cotterill P.J., Haslop J.M., Smith P.N., Tyson R.G. (1988). Fluorescent Compounds and Their Use. European Patent Application.

[22] Gizur T., Nagy A., Derzsi B. (2014). New Industrial Procedure for Production of a Sulfonamide Derivative. Hungarian Patent Application.

[23] Boultif A., Louer D. (1991). Indexing of powder diffraction patterns for low-symmetry lattices by the successive dichotomy method. J. Appl. Crystallogr..

[24] David W.I.F., Shankland K., van de Streek J., Pidcock E., Motherwell W.D.S., Cole J.C. (2006). DASH: A program for crystal structure determination from powder diffraction data. J. Appl. Cryst..

[25] Higashi T. (1998). NUMABS rev. 2002.

[26] Rigaku/MSC Inc (2008). CrystalClear SM 1.4.0.

[27] Burla M.C., Caliandro R., Carrozzini B., Cascarano G.L., Cuocci C., Giacovazzo C., Mallamo M., Mazzone A., Polidori G. (2015). Crystal structure determination and refinement via SIR2014. J. Appl. Crystallogr..

[28] (2013). SHELXL, version 2013.

[29] Farrugia L.J. (2012). WinGX and ORTEP for Windows: An Update. J. Appl. Crystallogr..

[30] Spek A.L. (2003). Single-crystal structure validation with the program PLATON. J. Appl. Crystallogr..

[31] Macrae C.F., Edgington P.R., McCabe P., Pidcock E., Shields G.P., Taylor R., Towler M., van De Streek J. (2006). Mercury: Visualization and analysis of crystal structures. J. Appl. Crystallogr..

[32] Westrip S.P. (2010). publCIF: Software for editing, validating and formatting crystallographic information files. J. Appl. Crystallogr..

[33] Spackman M.A., Jayatilaka D. (2009). Hirshfeld surface analysis. CrystEngComm.

[34] Spackman M.A., McKinnon J.J. (2002). Fingerprinting intermolecular interactions in molecular crystals. CrystEngComm.

[35] McKinnon J.J., Spackman M.A., Mitchell A.S. (2004). Novel tools for visualizing and exploring intermolecular interactions in molecular crystals. Acta Cryst. B.

[36] Shöniger W. Analytical procedures for the flask combustion method. Proceedings of the International Symposium on Microchemistry.

[37] https://github.com/ccdc-opensource/dash/releases.

[38] Markvardsen A.J., David W.I.F., Johnson J.C., Shankland K. (2001). A probabilistic approach to space-group determination from powder diffraction data. Acta Crystallogr. Sect. A.

[39] Bernstein J. (2002). Polymorphism in Molecular Crystals.

[40] Castle R.N., Witt N.F. (1946). The Polymorphism of Sulfapyridine. J. Am. Chem. Soc..

[41] Bar J., Bernstein J. (1985). Conformational Polymorphism VI: The Crystal and Molecular Structures of Form II, Form 111, and Form V of 4-Amino-N-2-pyridinylbenzenesulfonamide (Sulfapyridine). J. Pharm. Sci..

[42] Bernstein J. (1988). Polymorph IV of 4-amino-N-2-pyridinylbenzenesulfonamide (sulfapyridine). Acta Cryst..

[43] Gelbrich T., Threlfall T.L., Bingham A.L., Hursthouse M.B. (2007). Hursthouse, Polymorph VI of sulfapyridine: Interpenetrating two- and three-dimensional hydrogen-bonded nets formed from two tautomeric forms. Acta Cryst..

[44] Pratt J., Hutchinson J., Stevens C.L.K. (2011). Sulfapyridine (polymorph III), sulfapyridine dioxane solvate, sulfapyridine tetrahydrofuran solvate and sulfapyridine piperidine solvate, all at 173 K. Acta Cryst..

[45] Bora P., Saikia B., Sarma B. (2020). Oriented Crystallization on Organic Monolayers to Control Concomitant Polymorphism. Chem. Eur. J..

[46] Sovago I., Gutmann M.J., Grant Hill J., Senn H.M., Thomas L.H., Wilson C.C., Farrugia L.J. (2014). Experimental Electron Density and Neutron Diffraction Studies on the Polymorphs of Sulfathiazole. Cryst. Growth Des..

[47] Hughes D.S., Hursthouse M.B., Threlfall T., Tavener S. (1999). A new polymorph of sulfathiazole. Acta Crystallogr. Sect. C Cryst. Struct. Commun..

[48] Gelbrich T., Hughes D.S., Hursthouse M.B., Threlfall T.L. (2008). Packing similarity in polymorphs of sulfathiazole. CrystEngComm.

