# Supplementary material for: The Polymorphism of Orlyum White 520T, an Ultraviolet Luminescent Security Ink
Source: Molecules. 2025 Apr 8;30(8):1671. doi: 10.3390/molecules30081671 (PMC12029532; doi:10.3390/molecules30081671)
Supplement: Supplementary file 1 [file molecules-30-01671-s001.zip › molecules-3522465-supplementary.pdf]

Supplementary Materials

The polymorphism of Orlyum White 520T, an UV luminescent security ink

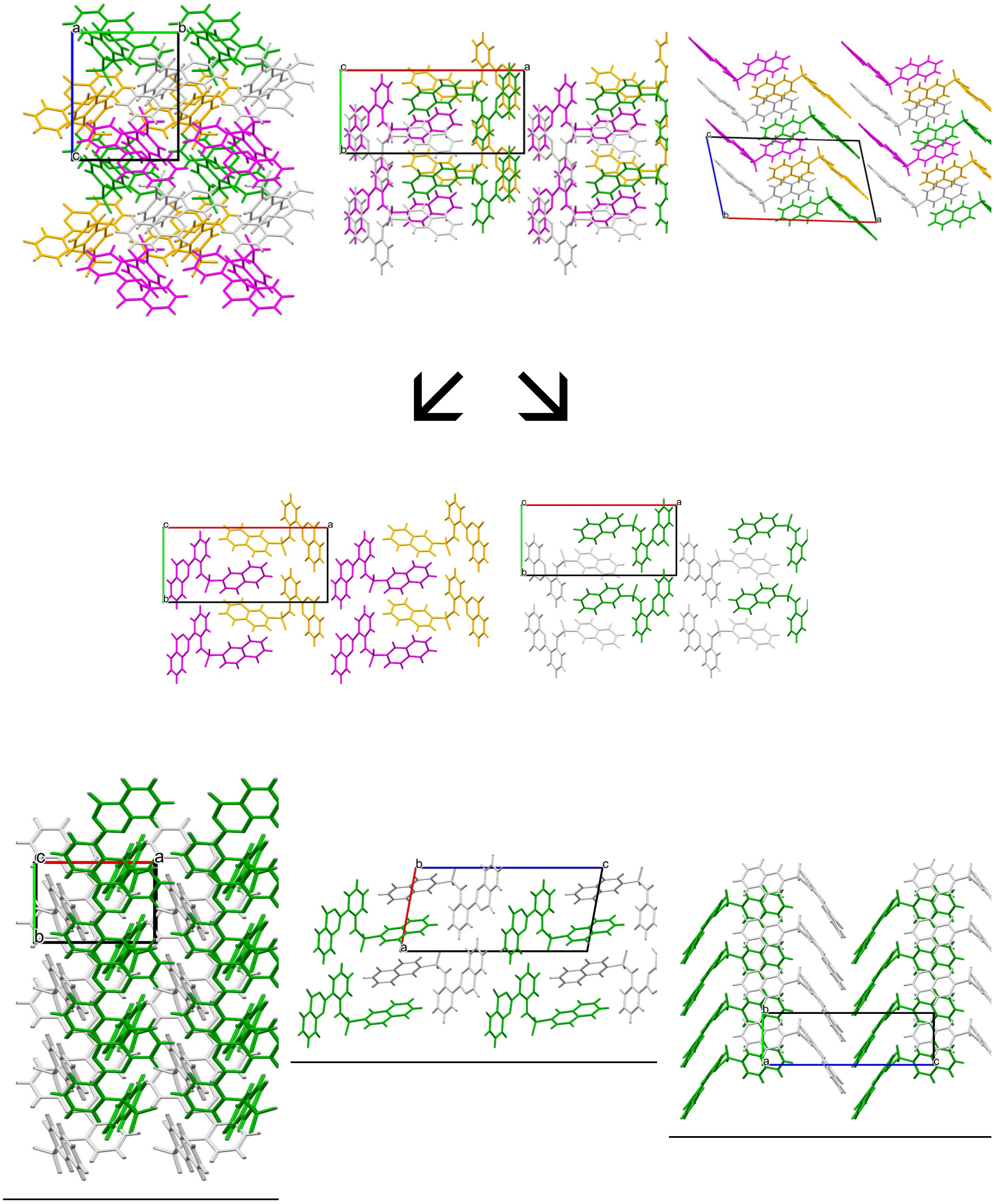

Figure S1 Packing arrangements of the molecules in Form II viewed from the crystallographic directions *a, c* and *b* (first raw). Molecules in the two layers of Form II viewed from the crystallographic *c* direction (second raw). Packing arrangements of the molecules in Form III viewed from the crystallographic directions *c, b* and *a* (third raw). Hydrogens are omitted for clarity. Molecules are coloured by symmetry operation. Corresponding

molecular placements in Forms II and III: green (II) and white (III), purple (II) and green (III).

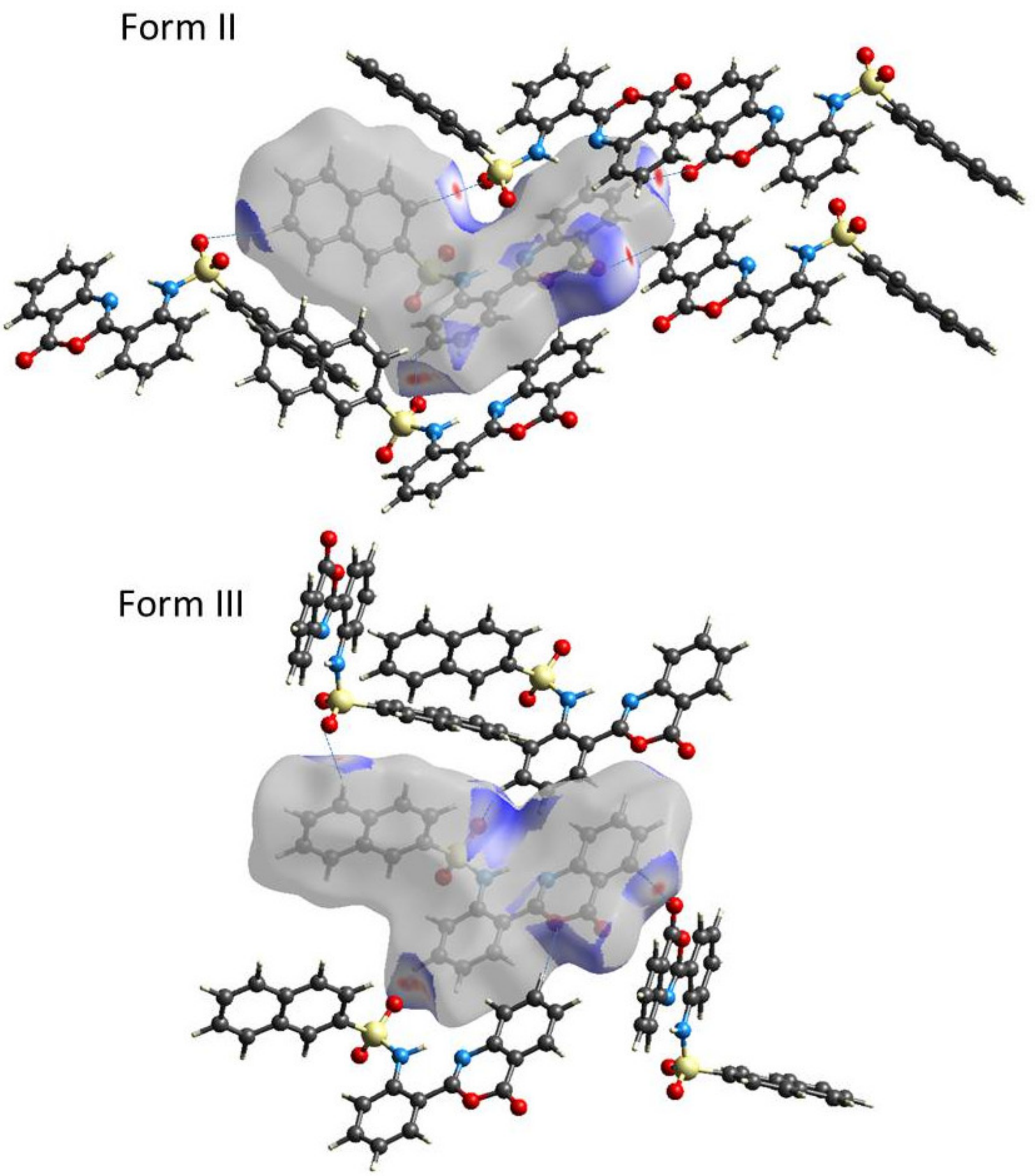

Figure S2. Comparison of the O…H interactions in Forms II and III of N-(2-(4-oxo-4H-benzo[d][1,3]oxazin-2-yl)phenyl)naphthalene- -2-sulfonamide howing the Hirshfeld surfaces. Neighbouring molecules associated with close contacts are shown.

Form II

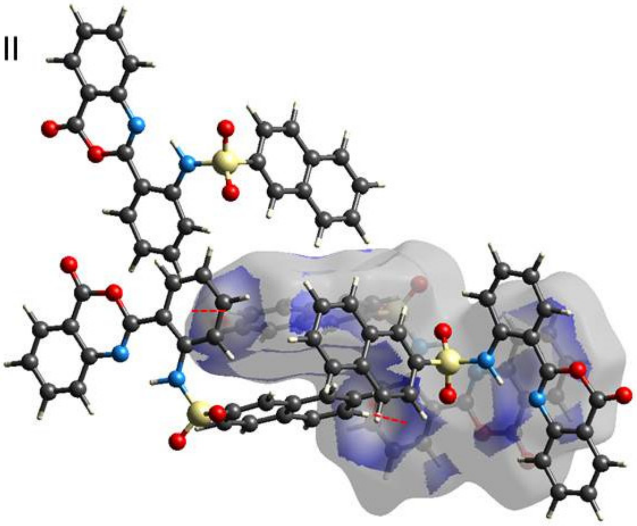

Form III

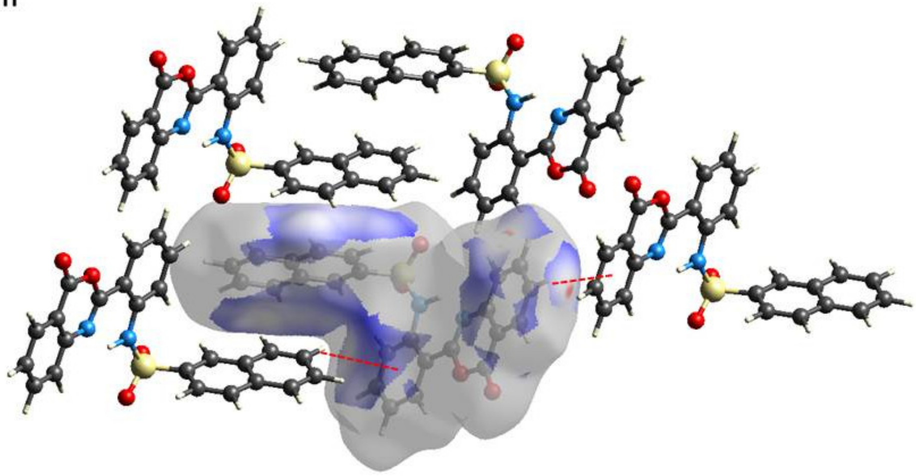

Figure *S*3. Comparison of the C…H interactions in Form II and III of N-(2-(4-oxo-4H-benzo[d][1,3]oxazin-2-yl)phenyl)naphthalene- -2-sulfonamide showing the Hirshfeld surfaces. Neighbouring molecules associated with close contacts are shown.

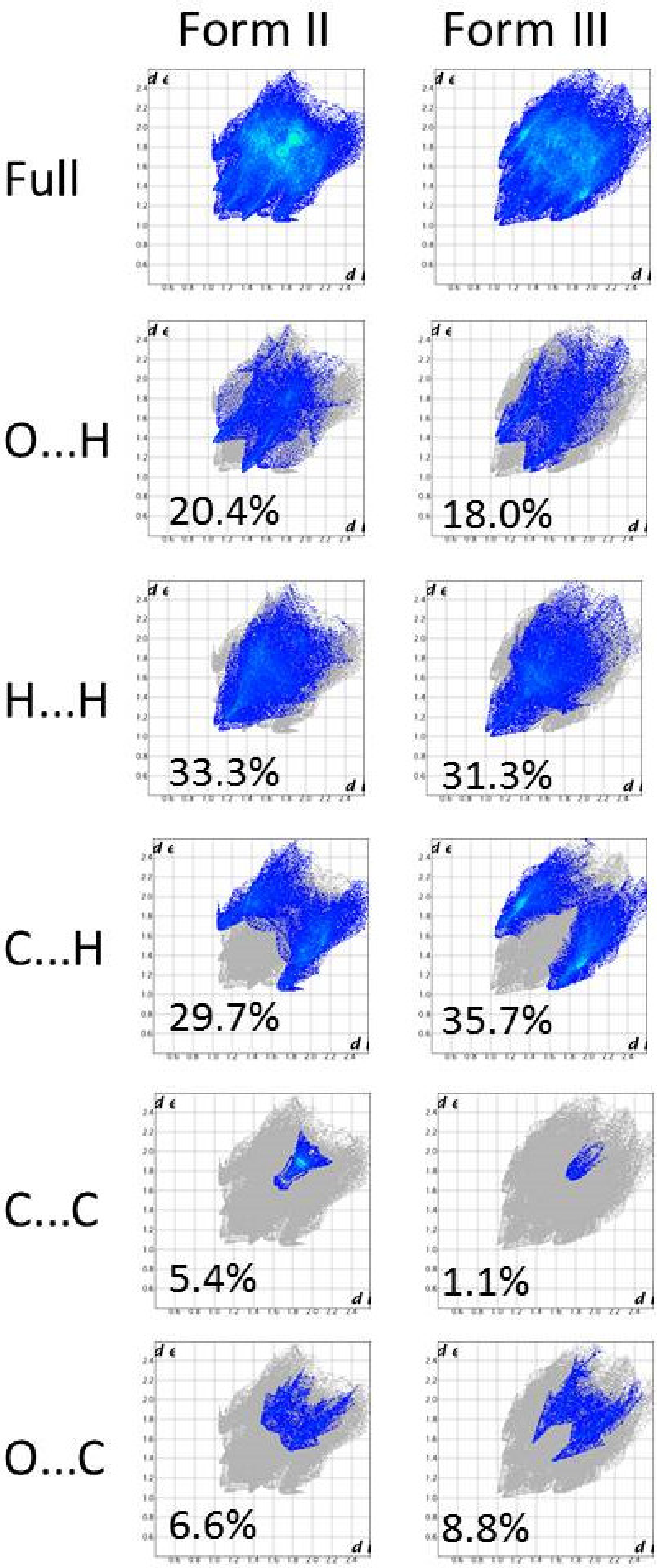

Figure S4. Contributions of specific pairs of atom-types to the full 2D fingerprint plots calculated in the crystals of Form II and Form III of N-(2-(4-oxo-4H-benzo[d][1,3]oxazin-2-yl)phenyl)naphthalene-2-sulfonamide.

Form II

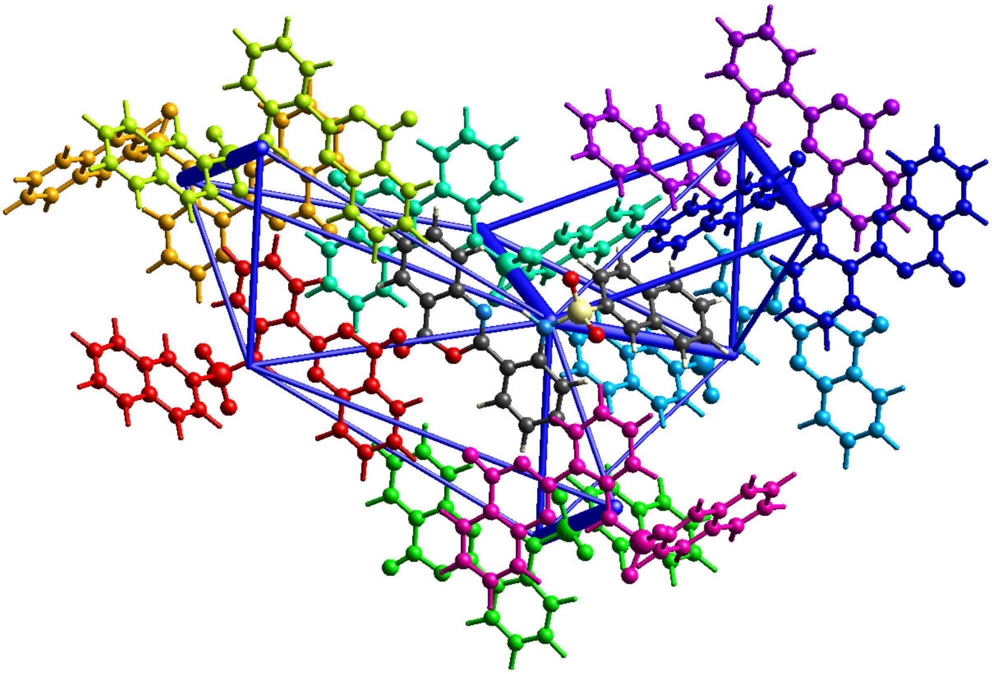

Form III

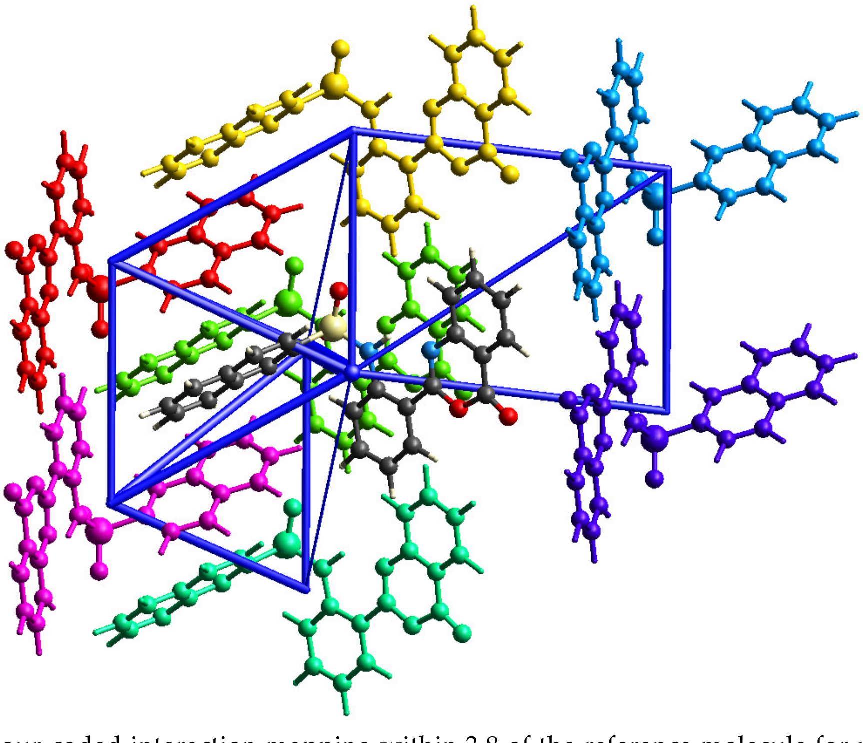

Figure *S*5. Colour-coded interaction mapping within 3.8 of the reference molecule for polymorphic crystals Form II and Form III with the obtained E_{tot} values between selected molecular pairs (calculated energy data are collected in Table *S*1).

Table S1. Colour-coded interaction energies calculated for polymorphic crystals of **Form II** and **Form III**

	N	Sym. op.	R	Electron Density	E_ele	E_pol	E_dis	E_rep	E_tot
**Form II**									
	1	-x, -y, -z	11.67	B3LYP/6-31G(d,p)	-4.0	-1.9	-23.5	6.9	-21.8
	2	-x, y+1/2, -z+1/2	15.31	B3LYP/6-31G(d,p)	-10.0	-2.7	-8.5	0.0	-20.0
	1	-x, -y, -z	13.40	B3LYP/6-31G(d,p)	0.0	-0.7	-19.2	0.0	-17.2
	1	x, y, z	8.88	B3LYP/6-31G(d,p)	-12.8	-5.6	-26.6	24.7	-25.6
	1	x, -y+1/2, z+1/2	6.02	B3LYP/6-31G(d,p)	-16.7	-7.2	-75.6	51.1	-57.2
	0	-x, -y, -z	9.40	B3LYP/6-31G(d,p)	-10.1	-1.7	-75.3	50.6	-46.2
	0	-x, y+1/2, -z+1/2	11.59	B3LYP/6-31G(d,p)	-9.6	-2.1	-31.8	21.2	-26.3
	0	-x, -y, -z	11.47	B3LYP/6-31G(d,p)	-3.5	-1.0	-14.4	5.1	-13.8
	1	x, -y+1/2, z+1/2	8.47	B3LYP/6-31G(d,p)	-2.7	-1.7	-22.4	7.2	-19.2
**Form III**									
	2	-x, y+1/2, -z	9.93	B3LYP/6-31G(d,p)	-9.7	-3.2	-30.9	17.6	-28.7
	1	x, y, z	8.78	B3LYP/6-31G(d,p)	-14.2	-6.7	-25.7	27.8	-25.2
	1	x, y, z	5.75	B3LYP/6-31G(d,p)	-8.7	-6.3	-78.3	40.9	-56.9
	0	x, y, z	10.49	B3LYP/6-31G(d,p)	-3.5	-0.6	-15.3	13.6	-9.0
	0	-x, y+1/2, -z	12.83	B3LYP/6-31G(d,p)	-4.1	-0.8	-14.1	0.0	-17.2
	0	-x, y+1/2, -z	10.78	B3LYP/6-31G(d,p)	-13.5	-3.4	-21.0	15.4	-25.6
	2	-x, y+1/2, -z	10.56	B3LYP/6-31G(d,p)	-7.9	-1.6	-44.6	28.8	-30.6

N is the number of pair(s) of interacting molecules with respect to the reference molecule, R is the centroid-to-centroid distance between the reference molecule and interacting molecules, Sym. op. is the symmetry operations with respect to the reference molecule, E_ele is the elecrostatic energy component, E_pol is polarization, E_dis is dispersion, E_rep is exchange-repulsion, and E_tot is the sum of the previous four energy values.
